# Supplementary material for: HIVprotI: an integrated web based platform for prediction and design of HIV proteins inhibitors
Source: J Cheminform. 2018 Mar 9;10:12. doi: 10.1186/s13321-018-0266-y (PMC5845081; doi:10.1186/s13321-018-0266-y)
Supplement: Supplementary file 2 — Additional file 2. Source code of HIVProtI web server. [file 13321_2018_266_MOESM2_ESM.zip › HIVprotI_Source-code/hivprotI/weka-3-6-6/doc/weka/datagenerators/ClusterDefinition.html]

ClusterDefinition


---


|  |  |  |
| --- | --- | --- |
| **Overview** \| **Package** \| **Class** \| **Tree** \| **Deprecated** \| **Index** \| **Help** \| **Weka's home** | |  |
| **PREV CLASS**   **NEXT CLASS** | **FRAMES**    **NO FRAMES**     **All Classes** |  |
| SUMMARY: NESTED \| FIELD \| CONSTR \| METHOD | DETAIL: FIELD \| CONSTR \| METHOD |  |


---


## weka.datagenerators Class ClusterDefinition

```
java.lang.Object
  weka.datagenerators.ClusterDefinition
```

**All Implemented Interfaces:**: java.io.Serializable, OptionHandler, RevisionHandler

**Direct Known Subclasses:**: SubspaceClusterDefinition

---

``` public abstract class ClusterDefinition extends java.lang.Object implements java.io.Serializable, OptionHandler, RevisionHandler ```

Ancestor to all ClusterDefinitions, i.e., subclasses that handle their
own parameters that the cluster generator only passes on.

**Version:**
:   $Revision: 1.5 $

**Author:**
:   FracPete (fracpete at waikato dot ac dot nz)

**See Also:**: Serialized Form

---

**Constructor Summary**

`ClusterDefinition()`
  
          initializes the cluster, without a parent cluster (necessary for GOE)

`ClusterDefinition(ClusterGenerator parent)`
  
          initializes the cluster


| **Method Summary** | |
| --- | --- |
| `abstract  java.lang.String[]` | `getOptions()`             Gets the current settings of the datagenerator BIRCHCluster. |
| `ClusterGenerator` | `getParent()`             returns the parent datagenerator this cluster belongs to |
| `java.lang.String` | `globalInfo()`             Returns a string describing this data generator. |
| `abstract  java.util.Enumeration` | `listOptions()`             Returns an enumeration describing the available options. |
| `java.lang.String` | `parentTipText()`             Returns the tip text for this property |
| `abstract  void` | `setOptions(java.lang.String[] options)`             Parses a list of options for this object. |
| `void` | `setParent(ClusterGenerator parent)`             sets the parent datagenerator this cluster belongs to |
| `java.lang.String` | `toString()`             returns a string representation of the cluster |

**Methods inherited from class java.lang.Object**

`equals, getClass, hashCode, notify, notifyAll, wait, wait, wait`

**Methods inherited from interface weka.core.RevisionHandler**

`getRevision`

**Constructor Detail**

### ClusterDefinition

```
public ClusterDefinition()
```

:   initializes the cluster, without a parent cluster (necessary for GOE)

---


### ClusterDefinition

```
public ClusterDefinition(ClusterGenerator parent)
```

:   initializes the cluster

    **Parameters:**: `parent` - the datagenerator this cluster belongs to


**Method Detail**

### globalInfo

```
public java.lang.String globalInfo()
```

:   Returns a string describing this data generator.

    :   **Returns:**: a description of the data generator suitable for displaying in the explorer/experimenter gui

---


### listOptions

```
public abstract java.util.Enumeration listOptions()
```

:   Returns an enumeration describing the available options.

    :   **Specified by:**: `listOptions` in interface `OptionHandler`
    :   **Returns:**: an enumeration of all the available options

---


### setOptions

```
public abstract void setOptions(java.lang.String[] options)
                         throws java.lang.Exception
```

:   Parses a list of options for this object.

    For list of valid options see class description.

    :   **Specified by:**: `setOptions` in interface `OptionHandler`
    :   **Parameters:**: `options` - the list of options as an array of strings **Throws:**: `java.lang.Exception` - if an option is not supported

---


### getOptions

```
public abstract java.lang.String[] getOptions()
```

:   Gets the current settings of the datagenerator BIRCHCluster.

    :   **Specified by:**: `getOptions` in interface `OptionHandler`
    :   **Returns:**: an array of strings suitable for passing to setOptions

---


### getParent

```
public ClusterGenerator getParent()
```

:   returns the parent datagenerator this cluster belongs to

    :   **Returns:**: the parent this cluster belongs to

---


### setParent

```
public void setParent(ClusterGenerator parent)
```

:   sets the parent datagenerator this cluster belongs to

    :   **Parameters:**: `parent` - the parent datagenerator

---


### parentTipText

```
public java.lang.String parentTipText()
```

:   Returns the tip text for this property

    :   **Returns:**: tip text for this property suitable for displaying in the explorer/experimenter gui

---


### toString

```
public java.lang.String toString()
```

:   returns a string representation of the cluster

    :   **Overrides:**: `toString` in class `java.lang.Object`
    :   **Returns:**: the cluster definition as string


---


|  |  |  |
| --- | --- | --- |
| **Overview** \| **Package** \| **Class** \| **Tree** \| **Deprecated** \| **Index** \| **Help** \| **Weka's home** | |  |
| **PREV CLASS**   **NEXT CLASS** | **FRAMES**    **NO FRAMES**     **All Classes** |  |
| SUMMARY: NESTED \| FIELD \| CONSTR \| METHOD | DETAIL: FIELD \| CONSTR \| METHOD |  |


---
